# Exploratory pilot study of a virtual support group for stroke caregivers

**DOI:** 10.3389/frhs.2026.1847116

**Published:** 2026-07-14

**Authors:** Kathryn Sabo, Alexandra Podell, Kimberly Erler

**Affiliations:** 1 Department is School of Nursing, MGH Institute of Health Professions, Boston, MA, United States; 2 Spaulding Rehabilitation Hospital Boston, Boston, MA, United States; 3 Department of Occupational Therapy, MGH Institute of Health Professions, Boston, MA, United States

**Keywords:** caregiver, health promotion, implementation science, interprofessional, support group, stroke

## Abstract

**Introduction:**

Despite advances in acute stroke care, long-term disability remains prevalent, placing substantial demands on informal caregivers. As structured rehabilitation in the chronic phase of stroke wanes, caregivers face evolving emotional, social, and logistical challenges with limited support, yet accessible and sustainable models of care remain limited.

**Methods:**

This implementation-informed exploratory pilot study evaluated the feasibility, acceptability, and preliminary outcomes of a virtual stroke caregiver support group.

**Results:**

Five participants attended weekly virtual sessions over six weeks facilitated by healthcare professionals. The virtual format addressed accessibility barriers frequently experienced by caregivers including travel time and distance, money, and caregiving responsibilities. Participants had moderate levels of loneliness and some engagement in self-care activities. Participants reported high satisfaction with and perceived benefits of the intervention, noting improved coping, increased confidence in the caregiving role, and validation for their lived experience as a caregiver.

**Discussion:**

The results underscore the need for inclusive, scalable caregiver interventions and greater healthcare provider engagement in caregiver support. Furthermore, the findings of this study support the feasibility and acceptability of a virtual caregiver support group in community-based settings and its potential to promote caregiver well-being.

## Introduction

While advances in acute stroke care have improved survival rates, stroke remains one of the leading causes of serious, long-term disability worldwide with an estimated 101 million people living with the effects of stroke globally, and 795,000 individuals experiencing a stroke each year in the United States (1, 2). The sudden onset of stroke leaves survivors and their families unprepared for its profound and often enduring consequences across both acute and chronic phases of recovery. Consistent with consensus frameworks in stroke recovery research, the chronic phase is considered the period beyond the first six months post-stroke, when rapid spontaneous biological recovery has largely diminished and longer-term adaptation, compensation, and support needs predominate (3). The range and extent of impairments is complex and varies based on location, type, and size of the stroke with potential impact on mobility, cognition, emotional health, and communication (2). With progress in neurorehabilitation failing to match the advancement in acute management of stroke, many individuals live longer with substantial disability and sustained dependence in basic activities of daily living, social participation, work, and community engagement (4). The high rates of disability post stroke underscore the need for long-term care and support (2). In the United States, underfunded, fragmented, and costly healthcare systems often fail to meet these ongoing needs, leaving families to assume the primary role of informal caregivers (5).

Stroke survivors receive specialized attention in the acute and subacute phases of recovery, when rehabilitation is intensive and highly structured. Yet as professional services taper, informal caregivers assume increasing responsibility for day-to-day support, enabling survivors to remain at home and engaged in their communities (6, 7). The caregiving role extends well beyond physical assistance, demanding sustained emotional support, advocacy, and continual problem-solving in everyday life (4). Caregiver well-being is directly tied to survivor outcomes, influencing functional recovery, emotional adjustment, and community participation (5, 8). Despite this central role, caregivers are too often regarded as peripheral to rehabilitation, leaving their needs systematically overlooked (4, 5).

Informal caregiver needs evolve across the stroke recovery trajectory, reflecting changes in the functional needs of the stroke survivor, the caregiver's own adjustment, and the interpersonal relationship between the survivor and the caregiver (8). This evolution leaves caregivers navigating complex, long-term responsibilities with little guidance, which can exacerbate their sense of isolation and burden (5). Emerging research highlights that long-term caregivers often report greater emotional distress, social isolation, and unmet informational needs than those in earlier phases, emphasizing the need for pragmatic, tailored approaches (8).

Despite decades of research demonstrating the benefits of informal caregiver interventions, translation into accessible and sustainable models of care has lagged considerably. Evidence supports the effectiveness of caregiver support interventions (9, 10), but the integration of these findings into routine practice is often slow and inconsistent, reflecting a persistent research-to-practice gap. Most interventions have been developed and tested during the acute or subacute phases of stroke recovery, typically within controlled research environments, limiting adaptation for long-term, community-based caregiving contexts (11, 12). Consequently, their feasibility, acceptability, and sustainability in real-world settings remain insufficiently understood. As a result, caregivers in the chronic phase, who often experience ongoing stress, social isolation, and declining well-being, have limited access to structured, evidence-informed support. Implementation-informed approaches may help accelerate the translation of effective interventions into practice by focusing on feasibility, acceptability, and sustainability within routine care settings (13). Accordingly, this study aims to address this gap by examining the real-world implementation of a caregiver-focused support intervention.

The primary aim of this exploratory study was to evaluate the feasibility and acceptability of a virtual support group for informal caregivers in a pro-bono academic center. In the absence of evaluating intervention effectiveness, this study explored who participated, their level of engagement, and their feedback regarding acceptability.

## Methods

### Design

This was a mixed-method exploratory study that utilized survey design to assess feasibility and acceptability of the virtual caregiver support group. Feasibility was defined as the ability to deliver the intervention as planned, including session delivery, attendance, retention, and completion of study procedures. Acceptability was defined as participants’ satisfaction with the program content, structure, and facilitation, assessed through post-program satisfaction ratings and open-ended feedback. Preliminary outcomes referred to descriptive pre to post-intervention changes in caregiver loneliness, examined to inform future research rather than to test efficacy. Quantitative data included feasibility indicators (attendance and retention), descriptive survey measures, and pre to post-intervention loneliness scores, while qualitative data were derived from open-ended satisfaction survey responses analyzed thematically.

The Stroke Caregiver Virtual Support Group was based at the Impact Practice Center, a community-based pro bono learning center at the MGH Institute of Health Professions. The Institute, a graduate school in the Northeast affiliated with the Mass General Brigham healthcare system, prepares future health professionals. The support group was facilitated by an occupational therapist and a registered nurse. The program was offered at no cost to all caregivers, independent of their insurance coverage. The program aimed to create a safe, supportive environment where participants could share their experiences, explore coping strategies, and receive affirmation of their caregiving roles. Session content combined elements of psychoeducation, skill-building, and psychosocial support. The session content was evidence-based and tailored specifically to the needs of stroke caregivers. Facilitators utilized a structured session outline and standardized educational resources to support consistency across group sessions. Each group met virtually for 60 min per session over a six-week period. Delivering the sessions through a secure online platform helped minimize common barriers such as transportation challenges and the need to balance caregiving responsibilities.

Each session was led by an occupational therapist and registered nurse. The facilitators were responsible for opening and closing the group, establishing expectations for participation, guiding discussion, and ensuring that all caregivers had space to contribute. Before each meeting, participants received an email with a secure link to join the virtual support group. These messages also offered assistance with navigating the online platform, with the goal of increasing participants’ comfort with the technology and reducing potential barriers to access.

**Figure 1:**
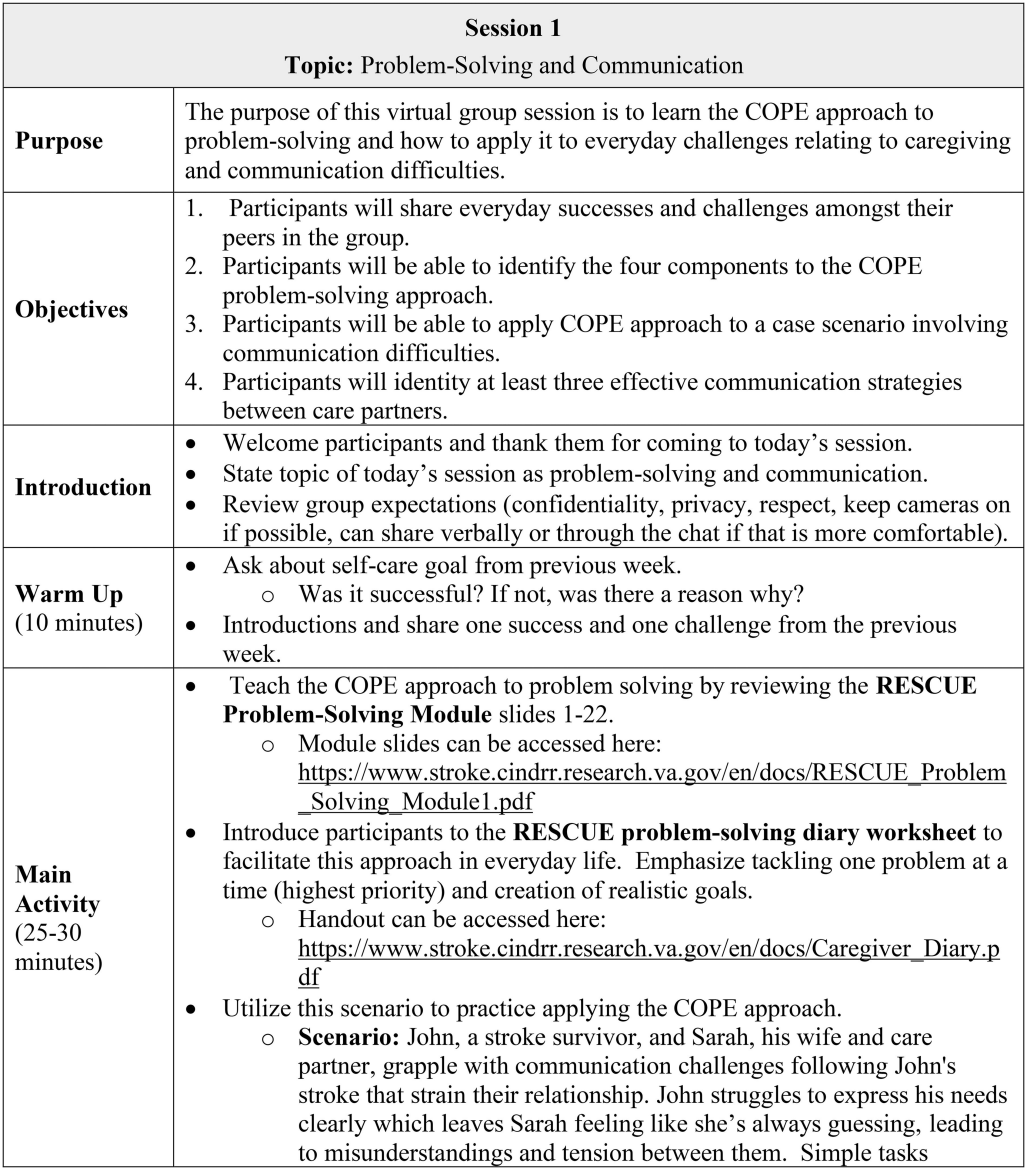
Session description and detailed outline.

Sessions opened with an orientation to platform features, a review of shared expectations, and brief introductions. Confidentiality was emphasized as a central component of group participation, with all attendees agreeing not to disclose information discussed outside the group. The facilitators then introduced a topic, encouraging caregivers to reflect on and share their experiences. Throughout the discussion, facilitators used active listening techniques and periodically summarized emerging themes to maintain clarity and foster group cohesion. When relevant, mindfulness-based stress-management strategies and health education were integrated. Figure 1 describes a typical weekly session. Given the sensitive nature of caregivers’ experiences, facilitators retained the ability to intervene if a participant showed signs of distress. At the close of each session, caregivers were invited to reflect on key takeaways, encouraged to attend the following meeting, and reminded of the availability of free individual counseling services through the Impact Practice Center.

**Figure:**
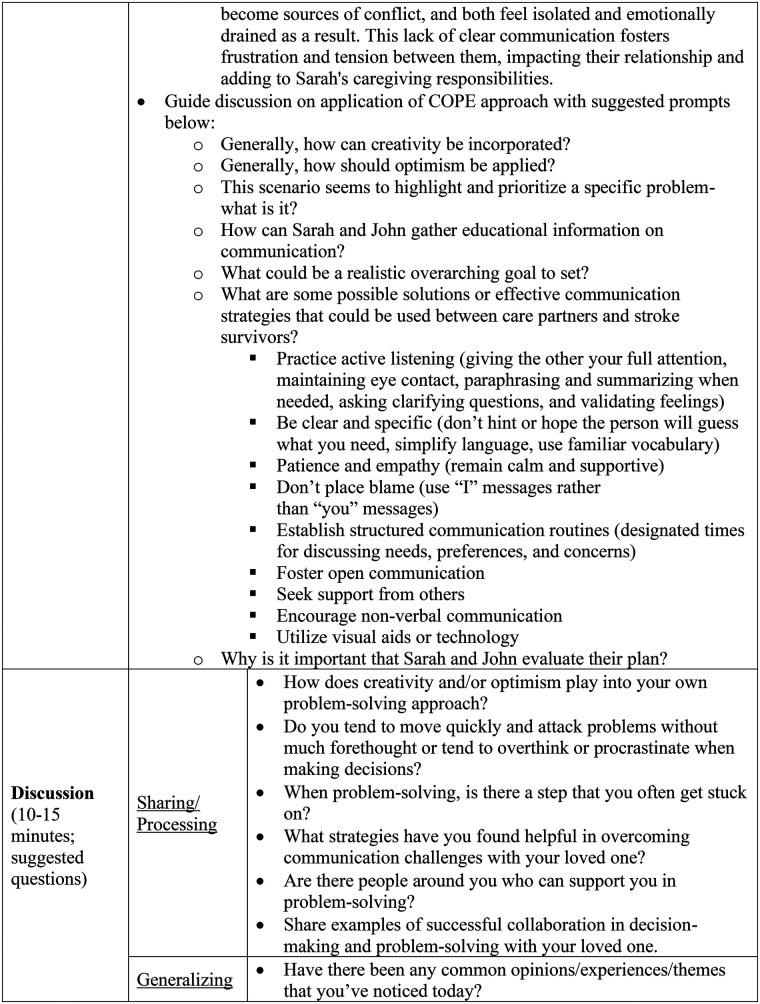


**Figure:**
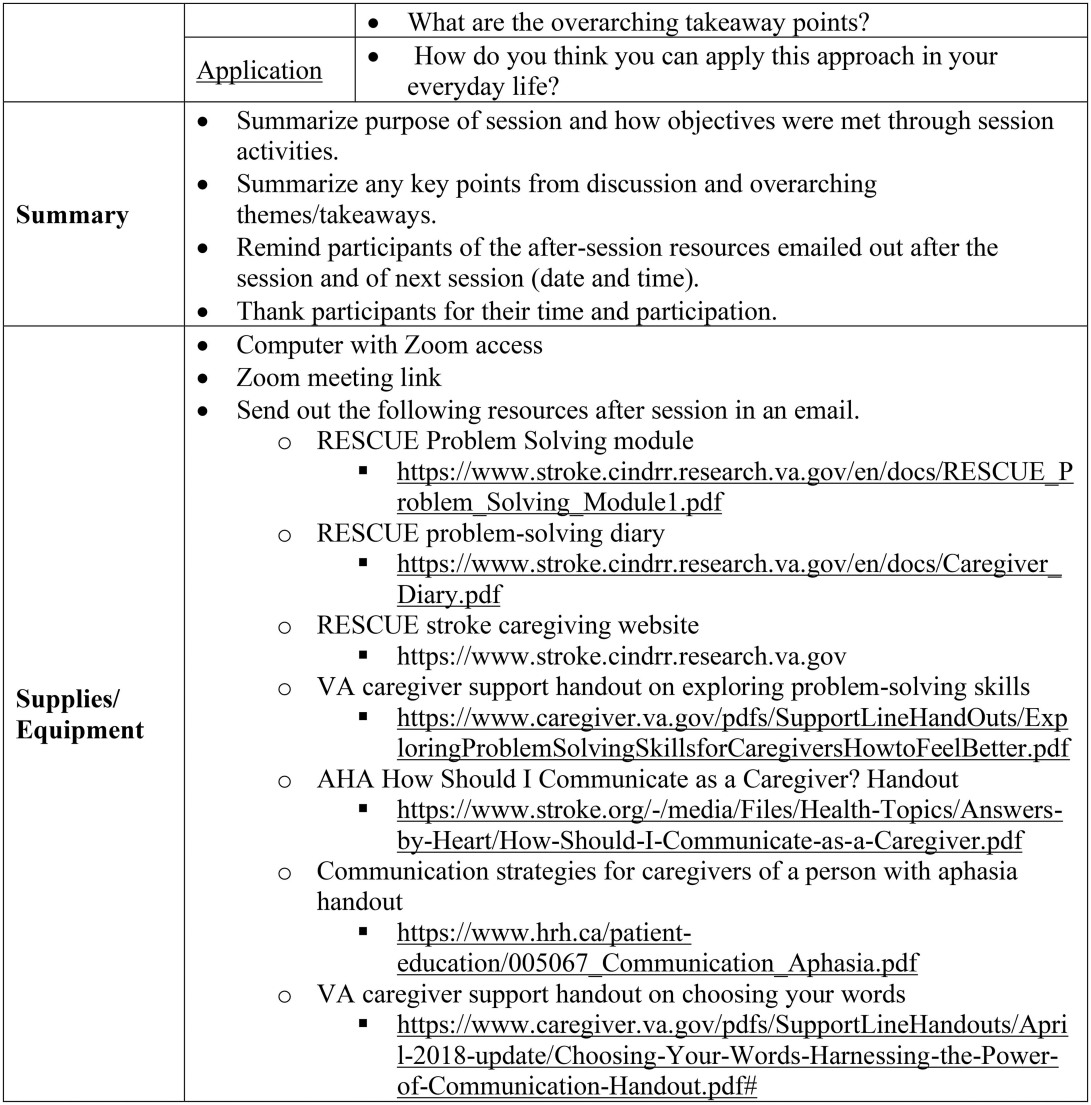


Participant recruitment occurred through collaborations with several stroke community organizations that endorsed the support group including neurology medical practices and adaptive exercise programs. Organizations facilitated recruitment by sharing registration information and schedule details via posted flyers and email communications. Eligibility criteria consisted of the following: participants had to identify as a caregiver to a chronic stroke survivor (defined as ≥6 months post-stroke), reside in the state of Massachusetts, and be able to sign participation consent forms required of the pro bono academic center.

Competing demands associated with caregiving often limit caregivers’ ability to consistently engage in self-care activities (14), a concern frequently expressed by participants during enrollment. Considering the limited availability of caregiver-focused interventions and the well-documented benefits of self-care, individuals were not excluded based on anticipated attendance challenges. Participants were not required to disclose a reason for their absence. To better accommodate caregivers’ variable schedules, a rolling enrollment approach was adopted, allowing participants to enter the program at any point within the session cycle.

### Measurements

To assess program feasibility, emphasizing potential facilitators and barriers, descriptive measures of the caregiver participants were collected. This included demographics, level of engagement in self-care, and participation metrics.

### Caregiver self-care assessment

A baseline descriptive measure of engagement in self-care was assessed using the “How Well Do You Take Care of Yourself?” tool. This tool utilizes a five-point Likert scale to determine how frequently different statements relating to self-care practices and coping strategies applied to them (15). Higher scores reflect decreased engagement in self-care and risk for potential health problems. All scores were self-reported and recorded anonymously through an online platform. The self-care assessment was collected at baseline to descriptively characterize caregiver well-being and contextualize feasibility findings and was not intended as an intervention outcome.

Program records were maintained. Attendance was tracked for each session to document participation patterns and overall caregiver engagement.

### Level of loneliness

Loneliness was assessed pre and post intervention using the original version of the UCLA Loneliness Scale (16). This instrument evaluates the level of loneliness using 20 items. Each item is rated on a scale ranging from 1 (*never*) to 4 (*always*), and items are summed to provide a total score ranging from 20 to 80. Scores were categorized into low, moderate, and high levels using distribution-based thresholds. Scores below 40 (lower 25th percentile) were classified as low loneliness, scores between 40 and 59 (middle 50%) as moderate loneliness, and scores of 60 or higher (upper 25th percentile) as high loneliness (16). Scores were self-reported and recorded anonymously through an online platform.

Assessment of program acceptability occurred by exploring caregiver satisfaction with the program via an electronic satisfaction survey.

### Client satisfaction survey

A post-program satisfaction survey was utilized to evaluate participants’ satisfaction with the program including content, design, and group facilitators. The 23-item survey included five-point Likert scales as well as open-ended questions. Participants rated their satisfaction with the program on a five-point Likert scale from “strongly disagree” to “strongly agree.” Open ended questions sought to examine what participants found most beneficial from the program and feedback on what to change. The survey was also delivered through an online platform and scores were recorded anonymously.

### Data collection

Participants completed the loneliness scale and self-care assessment prior to the first support group meeting. The loneliness scale and the client satisfaction survey were completed after the last support group meeting.

### Data analysis

Data analysis was performed using IBM SPSS version 28. Quantitative data were analyzed using descriptive statistics. Frequency, mean, standard deviation, and range were used to summarize participant characteristics, feasibility indicators, loneliness scores, self-care engagement, and client satisfaction ratings. Pre and post-intervention loneliness scores were summarized descriptively to examine patterns of change over time. Given the pilot nature of the study and small sample size, analyses were exploratory and not intended to test intervention efficacy.

Open-ended responses from the post-program satisfaction survey were analyzed using an inductive thematic analysis approach. Two researchers independently analyzed responses to the open-ended items from the client satisfaction survey following Braun and Clarke's (17) six-phase framework: (1) familiarization with the data, (2) generating initial codes, (3) searching for themes, (4) reviewing themes, (5) defining and naming themes, and (6) producing the final report (18). Codes were developed inductively from the data. The researchers met iteratively to compare coding decisions, reconcile discrepancies through consensus, and refine thematic definitions.

### Ethics statement

This study was deemed exempt from ethics approval or research consent procedures by the Mass General Brigham Institutional Review Board (MGB IRB). MGB IRB viewed the study as a program evaluation and improvement.

## Results

### Participants

Five informal caregivers registered to participate in the support group. Eighty percent (*n* = 4) were female and 20% (*n* = 1) were male. Eighty percent (*n* = 4) of participants identified as White, and the remaining participant (*n* = 1) preferred not to disclose race/ethnicity. Length of time in the caregiver role varied among participants with a mean of 1.6 years (SD = 0.49) and the median was two years. The mean score for engagement in self-care prior to the start of the support group was 26.00 (SD = 4.24). This value indicates participants are engaged in some degree of self-care but need some improvement.

### Feasibility outcomes

Feasibility findings reflected both recruitment feasibility and procedural feasibility of intervention delivery. Recruitment feasibility was limited, as only five clients registered to participate in the support group over six weeks despite outreach through multiple community organizations and efforts to reduce logistical participation barriers through a no-cost virtual format. In contrast, procedural feasibility was supported. All six planned sessions were delivered as scheduled. Attendance was consistent across sessions, with a mean of four participants per session (SD = 1) and a range of two to five participants. All participants who enrolled completed the pre and post-intervention loneliness measures. Session-by-session attendance is presented in Table 1.

**Table 1:** Session description and detailed outline.

Session #	Participants (A, B, C, D, E)
1	A, B
2	A, B, C
3	A, B, D, E
4	A, B, C, D, E
5	A, B, C, E
6	A, B, C, E

### Preliminary loneliness outcomes

A percentile-based interpretation is used to categorized results from the UCLA Loneliness Scale (16). Mean score on the loneliness scale prior to the start of the support group, of a maximum possible score of 80, was 50.40 (SD = 13.52). This result is in the 50 percentile and indicates a moderate level of loneliness (16). Following the 6 support group meetings, the mean loneliness score was in the fiftieth percentile at 44.00 (SD = 13.75), indicating a decrease in loneliness, yet remaining at moderate level among participants (16).

### Acceptability outcomes

#### Overall satisfaction

All participants reported satisfaction with the support group topics and delivery method. Participants strongly agreed that the group facilitators were knowledgeable, respectful, and active listeners. One caregiver reported, “the group leaders did a great job facilitating the group and bringing out some really helpful discussions.” Participants reported that they had interest in continuing with the program but hoped for additional caregiver participation. One participant share, “for me, I wish more caregivers had attended so there were more opportunities to learn from others.”

#### Benefits

Participants identified several benefits of the support group such as the ability to discuss different methods for stress management, practice techniques and strategies as a group, and explore practical applications of such strategies to daily routines as a caregiver. Additionally, participants determined that learning problem solving and communication skills were especially beneficial when navigating medical appointments and advocating for their care recipient. One participant reported, “I valued the focus on supporting me as a caregiver and learning ways to best advocate for my loved one.” For many, the support group provided validation and a sense of camaraderie for their lived experience as a stroke caregiver. Participants appreciated the safe space provided by the support group and the opportunity to reminisce about their life prior to the stroke and reflect on how their identity has been altered. One attendee reported, “it helps to know that there are other people dealing with similar issues and hearing ideas of how they deal with them.” Participants reported that selecting a topic for the last session truly individualized the experience.

## Discussion

This implementation-informed pilot study examined the feasibility, acceptability, and preliminary outcomes of a virtual support group for informal stroke caregivers within a pro-bono, academic center. Findings indicate that the virtual format was feasible to deliver and acceptable to participants, and that descriptive pre to post-intervention data suggest a potential reduction in caregiver loneliness. These are important outcomes given the pivotal role informal caregivers play in supporting stroke survivors’ ability to remain at home and engaged in their communities. The positive response to the virtual support group is consistent with prior evidence demonstrating that caregiver support groups can reduce distress and enhance resilience (18, 19), while extending this literature by demonstrating feasibility and acceptability within a pro bono, community-based setting.

Although procedural feasibility was supported, recruitment feasibility remained challenging. One of the most salient findings of this pilot was the challenge of recruiting and sustaining participation among stroke caregivers. Despite clear evidence of caregiver stress, only five caregivers enrolled, reflecting the difficulty of engaging this population even when barriers such as cost and transportation were minimized through a free, virtual format. This reality underscores the tension between caregiver need and the pragmatics of participation including competing responsibilities, exhaustion, and limited bandwidth. These challenges may also reflect the fragmented nature of long-term health and social service systems following stroke, where caregivers often assume increasing responsibility with limited ongoing structured support. Moreover, all participants in this study identified as White, highlighting concerns about equitable reach and representation. Caregivers from underrepresented groups may experience unique structural barriers that further limit participation in supportive services. Applying an implementation-informed lens revealed that the central challenges to providing evidence-based caregiver support are not only in intervention content, but also in ensuring programs are designed to reach caregivers broadly, with attention to accessibility and inclusivity (11).

The reported benefits described in the client satisfaction surveys support the acceptability of this intervention. Participants reported that they benefitted from learning about and practicing coping strategies to address the stress they experience in their caregiver role. Navigating a challenging healthcare system, where oftentimes efforts are unsuccessful, is a regular responsibility of caregivers, and participants described gaining problem-solving and communication strategies that they perceived as helpful when advocating for their care recipient. Participants also emphasized the value of the group as a safe space to process feelings, receive validation of lived experiences, and develop camaraderie with others in similar roles.

The findings of this study indicate feasibility in implementing the virtual stroke caregiver support group. All planned sessions were delivered as scheduled, attendance was sustained across the six-week period, and all participants completed study procedures, supporting procedural feasibility. Participant feedback further highlighted key elements that may facilitate successful implementation. Participants shared that the group facilitators conveyed trust to allow caregivers to be vulnerable and authentic. Moreover, facilitators were proficient in active listening and conveying empathy to facilitate processing of feelings and validation of experiences. The virtual format may enhance implementation potential by accommodating caregivers who might otherwise face barriers related to travel time and distance, cost, caregiving responsibilities, and other health needs. Although virtual delivery may introduce challenges related to technology access and group connection, participants in this study reported feeling supported, validated, and connected to others with similar experiences. These findings suggest that meaningful peer support and group engagement may still be fostered within a virtual caregiver support format.

The results suggest a potential reduction in loneliness, as reflected in descriptive pre to post-intervention scores. Given the small pilot sample, these findings should be interpreted cautiously and are intended to inform future research rather than demonstrate efficacy. Future studies are warranted to further explore this finding. Healthcare providers are well positioned to positively influence health outcomes for stroke caregivers. A critical first step is acknowledging the unique and often unmet needs of this population and recognizing caregiver support groups as an essential intervention for promoting caregiver well-being. To effectively guide caregivers, providers require a clear understanding of the benefits of support groups and the core elements that contribute to their successful implementation. Caregivers also need assistance navigating and accessing available support resources, and providers have a responsibility to facilitate this connection. By fostering collaborative partnerships with caregivers and integrating support group referrals into routine practice, healthcare providers can meaningfully enhance caregiver engagement in self-care and mitigate the risk of adverse health outcomes.

Taken together, these findings begin to address the persistent research-to-practice gap that characterizes much of the caregiver intervention literature. Although a robust body of evidence supports the effectiveness of caregiver support interventions, these benefits often remain confined to research settings and are not consistently operationalized into real-world, sustainable care models. In this context, our implementation-informed exploratory study represents an important step toward translating established evidence into practice by examining how a caregiver support intervention can be feasibly delivered and accepted within a community-based, pro bono academic setting. By focusing on feasibility and acceptability, this study considers how this intervention may function in the contexts of where caregivers actually live and seek support. These findings underscore the need for continued how caregiver support interventions can be adapted and sustained within real-world community settings.

## Limitations

Several limitations of this study should be noted. The small, primarily White sample limits generalizability. The study relied on self-reported data, which showed that participants experienced loneliness and limited self-care. Stroke survivor clinical and functional characteristics (e.g., level of functional independence) were not collected in this pilot, limiting the ability to contextualize caregiver experiences relative to survivor impairment. Future work should explore strategies to increase diversity and incorporate more systematic pre and post-intervention outcome measures.

This pilot study provides preliminary evidence supporting the feasibility and acceptability of a virtual support group intervention for informal stroke caregivers. The findings suggest that such groups may serve as a supportive resource for caregivers in the chronic phase of stroke recovery. The virtual format offers an accessible solution, particularly for caregivers facing logistical or health-related barriers to in-person participation. Further exploration is need to examine the scalability of this intervention. These results underscore the importance of integrating caregiver-focused interventions into healthcare practice and highlight the need for further research to evaluate long-term outcomes and broader applicability. This study contributes to the growing body of literature advocating for structured, evidence-informed support for caregivers within community and clinical practice settings.

## Data Availability

The raw data supporting the conclusions of this article will be made available by the authors, without undue reservation.
