# Correction: Exploratory pilot study of a virtual support group for stroke caregivers

**DOI:** 10.3389/frhs.2026.1939989

**Published:** 2026-07-30

**Authors:** Kathryn Sabo, Alexandra Podell, Kimberly Erler

**Affiliations:** 1 Department is School of Nursing, MGH Institute of Health Professions, Boston, MA, United States; 2 Spaulding Rehabilitation Hospital Boston, Boston, MA, United States; 3 Department of Occupational Therapy, MGH Institute of Health Professions, Boston, MA, United States

**Keywords:** caregiver, health promotion, implementation science, interprofessional, support group, stroke

**Error in author list**

Changing author order

The order of authors in the author list of the published paper was erroneously given as:

Kathryn Sabo^1^*

Kimberly Erler^2^

Alexandra Podell^3^

The correct author list reads:

Kathryn Sabo^1^*

Alexandra Podell^3^

Kimberly Erler^2^

The original version of this article has been updated.

